# Low-altitude small target detection in sea clutter background based on improved CEEMDAN-IZOA-ELM

**DOI:** 10.1016/j.heliyon.2024.e26500

**Published:** 2024-02-18

**Authors:** Shang Shang, Jian Zhu, Qiang Liu, Yishan Shi, Tiezhu Qiao

**Affiliations:** Ocean College, Jiangsu University of Science and Technology, Zhenjiang, 212003, China

**Keywords:** CEEMDAN decomposition, RCMDE, AWT denoising, IZOA optimization algorithm, ELM

## Abstract

To effectively detect low-altitude small targets under complex sea surface environment, an innovative method has been developed. This method harnesses the chaotic characteristics of sea clutter and employs a combination of Adaptive Noise Complete Ensemble Empirical Modal Decomposition (CEEMDAN), Adaptive Wavelet Thresholding (AWT), and Polynomial Fitting Filtering (SG) for denoising sea clutter data. Subsequently, the Improved Zebra Optimization Algorithm-Extreme Learning Machine (IZOA-ELM) detector is utilized to identify low-altitude small targets amidst the sea clutter background. To begin, the CEEMDAN method is applied to disentangle the measured sea clutter data into a set of Intrinsic Mode Functions (IMFs). Afterwords, the Refined Composite Multiscale Dispersion Entropy (RCMDE) is computed for each individual IMF. This process categorizes the IMFs into three distinct components: noise-dominant, signal-noise mixture, and signal-dominant segments. The noise-dominate of IMF component is subjected to denoising through AWT, the signal-noise mixture of IMF components are processed using SG filtering, while the signal-dominant of IMF remains unaltered. The denoised sea clutter signal is reconstructed by concatenating the denoised and unprocessed IMFs. Based on the chaotic nature of sea clutter signals, first-order sea clutter data is transformed into high-dimensional data through phase space reconstruction. The initial weights and thresholds of the ELM are optimized through the IZOA to establish an optimal prediction model. This model is then used to detect small, low-altitude targets by analyzing the prediction error. The algorithm's effectiveness in noise removal is validated using IPIX and SPRR measured sea clutter data, demonstrating a significant improvement in the root mean square of prediction error (RMSE) by one order of magnitude after denoising compared to the pre-denoising state. Furthermore, we observed that the IZOA-ELM method can be effectively applied to detect small targets at low altitudes across various sea conditions. However, when the sea state is complex and greatly affected by the surrounding noise, an effective approach is to first employ CEEMDAN-AWT-SG to denoise the original signal, and then utilize IZOA-ELM for target detection.

## 1 Introduction

The detection of low-altitude small targets represents the initial challenge in low-altitude identification tracking and the monitoring of low-altitude airspace, as noted in Ref. [1]. Sea radar faces difficulty in detecting small, fast-moving, low-altitude targets due to their limited size and high speed. Therefore, they hold a crucial position in marine military detection, frequently employed for real-time reconnaissance of the surrounding sea conditions. To ensure an effective response to the threat presented by small, low-altitude targets to our coastal defense in China's extensive maritime domain, the establishment of dedicated maritime surveillance radar systems is imperative. Nonetheless, the real-world sea surface conditions are intricate and ever-changing. Electromagnetic waves emitted by maritime surveillance radar primarily irradiate the sea surface, with only a fraction directed towards the intended target. This phenomenon leads to the target's echo being obscured by intense sea clutter, significantly constraining the detection performance, as noted in Refs. [2,3]. In recent years, certain scholars have suggested that the identification of small, low-altitude targets can be achieved through the inherent characteristics of sea clutter. Undeniably, this proposition holds significant implications for the domain of sea surface monitoring and maritime defense [4].

Sea clutter refers to the echo reflected backward by the sea surface when it is irradiated by electromagnetic waves emitted from radar systems. Its formation and development are influenced by multiple factors, such as wind, tidal forces, ocean currents, and seafloor topography [5]. These factors render sea clutter inherently non-smooth, non-linear, and stochastic in nature. Scholars like He and his colleagues [6], have collectively termed these characteristics as the "three non-" attributes of sea clutter. Due to its "three non-" characteristics, the selection of a suitable adaptive signal decomposition method is of particular significance when it comes to reducing noise in sea clutter.

In 1998, a novel adaptive signal time-frequency processing method called Empirical Mode Decomposition (EMD) was introduced by Huang et al. from NASA [7]. EMD has proven to be highly influential in addressing nonlinear and non-smooth signals. Nevertheless, EMD decomposition has two inherent defects: mode mixing and the effect of endpoints. Considering the above defects, Huang E et al. [8] proposed Ensemble Empirical Modal Decomposition (EEMD) in 2009, which effectively solves the modal aliasing problem of EMD, while it brings time-consuming and residual noise problems in the reconstructed signals. Torres et al. [9] introduced CEEMDAN as an extension of the EMD technique. CEEMDAN incorporates concepts from methods such as adding Gaussian noise, and employs multiple superpositions for noise elimination. This innovative approach effectively addresses the limitations inherent in both EMD and EEMD. Lu [10] introduced a novel mixed model called CEEMDAN-GA-SVR. This model involves the decomposition of the original Center of Pressure (COP) signal into a set of simpler subsequences using CEEMDAN. Subsequently, it employs a Genetic Algorithm-Support Vector Regression (GA-SVR) approach to predict the values of these subsequences. Experimental results clearly demonstrate that the predictive accuracy achieved by the mixed model significantly outperforms that of both individual and integrated benchmark models. Ban et al. [11] used CEEMDAN for PM2.5 time series data and applied Long Short-Term Memory(LSTM), Back Propagation(BP), Autoregressive Integrated Moving Average Model(ARIMA), and Support Vector Machine(SVM) to each modal component to obtain a satisfactory integrated prediction model.

The pivotal factor when applying CEEMDAN for the denoising of sea clutter signals lies in accurately determining the boundary point between the signal and noise components. In the literature, several common methods for determination of the signal-to-noise boundary point in denoising sea clutter signals have been employed, including the correlation coefficient method [12], the average period and energy density product criterion [13], permutation entropy [14], and multiscale permutation entropy [15]. Contrary to the aforementioned methods, which often suffer from disadvantages such as prolonged computation times, limited feature extraction capabilities, and scene-specific limitations, RCMDE offers several distinct advantages. It excels in terms of rapid computation speed, robust stability, and the effective extraction of the intrinsic characteristics of signals.

In 1995, Haykin and his colleagues [16] made the pioneering discovery of the chaotic nature of sea clutter based on measured sea clutter data. This revelation captured significant attention within the community of sea clutter researchers, inspiring numerous investigations that harnessed this property to develop chaotic time series prediction models. Building on the chaotic properties of sea clutter, Xing and co-authors [17] put forth a method for weak signal detection using BP neural networks. Yan and teams [18] developed an LSTM network for the detection of small floating targets on the sea surface in the presence of chaotic backgrounds. As the need for enhanced detection accuracy and speed has grown, the conventional BP network has been progressively abandoned due to its simplistic architecture and limited self-learning capabilities. Even though LSTM satisfies the accuracy requirement, it takes a longer time to find the nonlinear relationship between the sea clutter data, which results in a slower training speed. The ELM network, a single-layer feedforward neural network based on randomization, offers rapid learning, high accuracy, and straightforward implementation [19]. It has emerged as a focal point of research in recent years. Tang and colleagues [20] conducted a denoised sea clutter prediction using GWO-optimized ELM in combination with ESMD-ICA denoising, which yielded significantly improved results compared to conventional BP predictions. Li [21] introduced an extreme learning machine that incorporates error self-correction and applied it to sea clutter prediction. The results demonstrated superior prediction performance compared to traditional autoregressive (AR) methods.

As for the existing prediction of sea clutter, two major problems exist (1) no consideration of the influence of noise around the measured sea clutter. (2) optimization of the prediction model. In this paper, its first innovations are in the area of sea clutter denoising: (i) using RCMDE to determine the signal-to-noise boundary point of IMF. (ii) In the case of noise-dominated IMF components, we employ an enhanced AWT method. This allows us to effectively attenuate noise while preserving the integrity of the original signals. The second key innovation lies in the IZOA optimization algorithm. This algorithm plays a pivotal role in achieving the utmost accuracy in the ELM prediction model. This accurate model is instrumental in the subsequent detection of small targets using prediction error analysis.

## 2 Sea clutter pre-processing algorithms

### 2.1 CEEMDAN decomposition

This approach is driven by the inherently nonlinear nature of sea clutter, which can be effectively decomposed into a series of IMFs through the use of CEEMDAN. These IMFs are arranged sequentially from high frequency to low frequency. High-frequency IMFs are prone to containing a higher proportion of noise from the original sea clutter, while medium-frequency IMFs combine both noise and the genuine sea clutter signals. In contrast, low-frequency IMFs are more likely to predominantly consist of pure sea clutter signals. Hence, precise categorization of these IMFs is a fundamental prerequisite for the successful denoising of sea clutter.

### 2.2 Refined composite multiscale dispersion entropy

The IMFs resulting from CEEMDAN decomposition of the original sea clutter sequence can be categorized into three groups: noise-dominated, signal-noise mixing, and signal-dominated. This categorization plays a central role in accurately delineating the boundaries between the signal and noise components, which is the core of sea clutter denoising. Azami [22] proposed the RCMDE method in 2017, which can identify complex nonlinear dynamics features in long sequences, so we use RCMDE as the signal-to-noise boundary point determination method in this paper. This is calculated in the following steps:

Step 1 Given that the original sequence is u and the length is S, divide u equally into τ segments and compute the mean value, then perform the coarse-graining operation to find the h th coarse-grained sequence as (1),

(1) $$\alpha_{h,\gamma }^{\tau }=\frac{1}{\tau }\sum_{n=h+\left(\gamma -1\right)\tau }^{h+\gamma \tau -1}u_{n}$$

Step 2 The above-desired coarse-grained sequence $\alpha =\left\{\alpha_{1},\alpha_{2},...,\alpha_{\frac{S}{\tau }}\right\}$ is mapped to

$\beta =\left\{\beta_{1},\beta_{2},...,\beta_{\frac{S}{\tau }}\right\}$, the exact equation is shown in (2),

(2) $$\beta_{\gamma }^{\tau }=\frac{1}{\sigma \sqrt{2\pi }}\int_{-\infty }^{\alpha_{\gamma }^{\tau }}e^{\frac{-{\left(t-\mu \right)}^{2}}{2\sigma^{2}}}dt$$

Where μ and σ are the mean and standard deviation of $\alpha_{\gamma }^{\tau }$, $\beta_{i}\in \left(0,1\right)$.

Step 3 Mapping $\beta_{\gamma }^{\tau }$ to {1,2,...,c}, recorded as z, the exact equation is shown in (3),

(3) $$z_{\gamma }^{c}=\left[c\cdot \beta_{\gamma }^{\tau }+0.5\right]$$

Where [⋅] is the rounding operation and c is the number of categories.

Step 4 Set the embedding dimension m, the time delay d, and the time series defined as (4),

(4) $$z_{i}^{m,c}=\left\{z_{i}^{c},z_{i+d}^{c},...,z_{i+\left(m-1\right)d}^{c}\right\}$$

Among them, $i=1,2,...,\frac{S}{\tau }-\left(m-1\right)d$.

Step 5 Compute the scattering pattern $\pi_{v_{0}v_{1}...v_{m-1}}\left(v=1,2,...,c\right)$ , recording $v^{m}=\left\{v_{0},v_{1},...,v_{m-1}\right\}$ , and if $v^{m}=z_{i}^{m,c}$ , the scattering pattern corresponding to $z_{i}^{m,c}$ is $\pi_{v_{0}v_{1}...v_{m-1}}$.

Step 6 Calculate the probability $p\left(\pi_{v_{0}v_{1}...v_{m-1}}\right)$ of each scattering pattern $\pi_{v_{0}v_{1}...v_{m-1}}$ , the exact equation is shown in (5),

(5) $$p\left(\pi_{v_{0}v_{1}...v_{m-1}}\right)=\frac{num\left(\pi_{v_{0}v_{1}...v_{m-1}}\right)}{\frac{S}{\tau }-\left(m-1\right)d}$$

Where $num\left(\pi_{v_{0}v_{1}...v_{m-1}}\right)$ represents the number of mappings of $z_{i}^{m,c}$ to $\pi_{v_{0}v_{1}...v_{n}}$.

Step 7 Calculate RCMDE at each scale τ, the exact equation is shown in (6),

(6) $$RCMDE\left(X,m,c,d,\tau \right)=-\sum_{\pi =1}^{c^{m}}\bar{p}\left(\pi_{v_{0}v_{1}...v_{m-1}}\right)In\bar{p}\left(\pi_{v_{0}v_{1}...v_{m-1}}\right)$$

where $\bar{p}\left(\pi_{v_{0}v_{1}...v_{m-1}}\right)=\frac{1}{\tau }\sum_{1}^{\tau }p_{k}^{\tau }$ is the average of the probabilities of the coarse-grained sequence $\alpha_{h,\gamma }^{\tau }$ in the scattering pattern π.

The parameter values in the RCMDE refer to the literature [23], where =2 , c=3 , d=1 , and τ=8 are derived. In addition, our analysis leads to the following conclusions regarding the RCMDE curves associated with different types of IMFs:①

For Noise-Dominated IMFs: The RCMDE curves exhibit a consistent monotonically decreasing pattern. A considerable variation is observed within these curves, signifying significant fluctuations. ②For Mixed Signal-Noise IMFs: The RCMDE curves for this category display one or more extreme points. ③For Signal-Dominated IMFs: The RCMDE curves in this case demonstrate a continual monotonically increasing trend. In contrast to noise-dominated IMFs, these curves exhibit minimal variation, suggesting a smoother and more stable behavior.

### 2.3 Adaptive wavelet thresholding denoising

Assuming that the original noise-containing signal is shown as (7):

(7) a(n)=b(n)+s(n),n=0,1,2,...,N−1

where a(n) is the noise-containing signal, b(n) is the original signal, s(n) is the noise signal, and N is the number of sampling points.

The critical aspect of denoising using wavelet threshold functions lies in the selection of both the threshold function and the threshold value. In this study, we propose an approach, inspired by prior works [24,25], which introduces adaptive threshold functions and thresholds. This adaptive approach is designed to align with changes in the number of decomposition layers, ensuring that the wavelet decomposition coefficients accurately respond to these changes. As a result, it facilitates a more precise filtering of noise at each layer, achieving maximum noise reduction. The exact adaptive function expression is as (8)、(9),

(8) $$\bar{w}=\left\{ \begin{array}{l} w-0.9\text{sgn}\left(w\right)\cdot \frac{\lambda^{m}}{{\left|w\right|}^{m-1}},\left|w\right|>\lambda \\ 0.1\text{sgn}\left(w\right)\cdot \frac{{\left|w\right|}^{9m+1}}{\lambda^{9m}},\left|w\right|\leq \lambda \end{array}\right.$$

(9) $$\lambda =\left(\left(median\left|w_{k}\right|\right)/0.6745\right)\cdot e^{2\cdot \log\frac{e+1}{e+k}}$$

where w is the original wavelet decomposition coefficients, are the coefficients processed by the threshold function, sgn(⋅) is the sign function, m is the adjustment factor, λ is the threshold gate, k is the number of decomposition layers, and $median\left(\left|w_{k}\right|\right)$ denotes the median of the wavelet coefficients of the k th layer of decomposition.

**Fig. 1:**
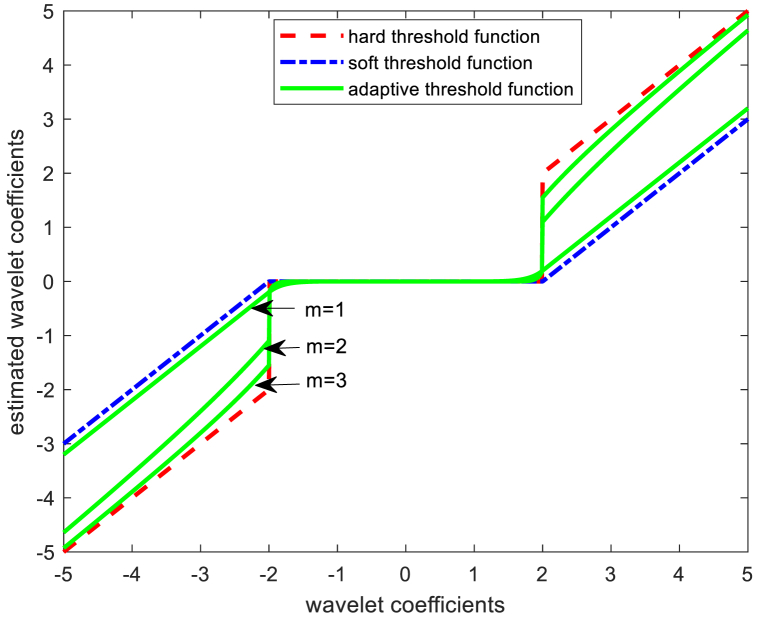
Adaptive threshold function.

The equation above clearly demonstrates that the adjustment of the parameter 'm' enables the threshold function to dynamically transition between the hard threshold function, as described in Ref. [26], and the soft threshold function, as discussed in Ref. [27]. This adjustment aligns the threshold function more closely with the hard threshold function within the fine scale of wavelet decomposition, effectively removing the majority of noise coefficients in this range. Simultaneously, it aligns the threshold function closer to the soft threshold function in the wider scale, thereby preserving the singular characteristics inherent in the signal. It can be clearly seen from Fig. 1: when the value of m is small, the function as a whole is biased towards a soft threshold function, and as the value of m becomes larger, it gradually converts to a hard threshold function.

## 3 Sea surface low altitude small target detection based on IZOA-ELM detector

### 3.1 Improved zebra optimization algorithm

The Zebra Optimization Algorithm is a novel intelligence optimization method introduced by Dehghani et al. in the year 2022, as documented in Ref. [28]. This algorithm effectively addresses real-world problem-solving and optimization challenges by simulating a range of behaviors inspired by natural phenomena, including behaviors observed in zebras, such as foraging and evading predators. While the Zebra Optimization Algorithm (ZOA) features a reduced parameter set and ease of operation, it is not immune to certain challenges, notably the risk of converging into local optima during practical optimization searches. In this paper, we enhance the ZOA through improvements in population initialization, foraging, and defense mechanisms. Furthermore, we apply this optimized algorithm to the initialization of parameters in the ELM) neural network. This enhancement significantly bolsters the stability of the ELM neural network model, thus laying the groundwork for precise predictions in the domain of sea clutter analysis.

(1) Population initialization: using the set of good points to initialize the population can effectively improve the diversity and uniformity of the population. The specific expressions are as (10):

(10) $$\begin{array}{l} r=\left(r_{1},r_{2},...,r_{N}\right),r_{j}=\mathrm{mod}\left(2\;\cos\left(\frac{2\pi i}{7}\right),1\right),1\leq j\leq N\\ P_{N}\left(i\right)=\left\{\left(r_{1}i_{1},r_{2}i_{2},...,r_{N}i_{N}\right)\right\},i=1,2,3,...,N\\ X_{i}^{j}=a_{j}+P_{N}\left(i\right)\left(b_{j}-a_{j}\right) \end{array}$$

where mod(·) is the residual function, $P_{N}\left(i\right)$ is the constructed set of good points of number N, $a_{j}$ is the lower bound of the current dimension, and $b_{j}$ is the upper bound of the current dimension.

(2) Within the foraging phase, population segmentation was determined by comparing individual zebra sizes with the mean fitness values of the population, which are expressed as (11):

(11) $$\varepsilon =\frac{1}{N}\sum_{i=1}^{N}fitness\left(i\right)$$

Where ε denotes the average fitness value of the population and N is the population size.

(12) $$x_{i,j}^{new,p1}=\left\{ \begin{array}{l} x_{i,j}+r\cdot \left(PZ_{j}-I\cdot x_{i,j}\right),fitness\left(i\right)\leq \varepsilon \\ x_{i,j}+r\cdot \left(x_{j}^{rand}-I\cdot x_{i,j}\right),fitness\left(i\right)>\varepsilon \end{array}\right.$$

In equation (12), $x_{i,j}^{new,p1}$ denotes the updated zebra position, $x_{i,j}$ denotes the original zebra position, $PZ_{j}$ denotes the pioneer zebra position, $x_{j}^{rand}$ denotes the random zebra position, r is a random number between [0,1], and I is a random value belonging to the set {1,2}.

(3) The acceleration of the algorithmic optimization search process during the defense phase is facilitated by integrating an adaptive probability threshold. This threshold assists in the determination of whether an escape or an attack strategy should be executed, which is expressed as (13):

(13) $$adaptive\_p=1-\frac{1}{4}\left(\frac{t}{T}+3\cdot {\left(\frac{t}{T}\right)}^{3}\right)$$

Where t represents the current number of iterations and T represents the maximum number of iterations.

(14) $$\left\{ \begin{array}{l} if\;p\leq \text{adaptive}\_p\to \text{Execute}\;\text{escape}\;\text{strategy}\\ if\;p>\text{adaptive}\_p\to \text{Execute}\;\text{attack}\;\text{strategy} \end{array}\right.$$

In equation (14), where p is a random number between [0,1] in the above equation.

In the case of a zebra executing the escape strategy, a shift from a linear to a nonlinear reduction in the adaptive step control factor is implemented to better align with the zebra's actual running trajectory, and the specific expression is as (15):

(15) $$\alpha =1-e^{\left[\left(\frac{t}{T}-1\right)*\left(\frac{T}{t-1}\right)\right]}$$

(4) In both the foraging and defense phases, the acceleration of convergence speed and enhancement of convergence accuracy across the entire zebra population are sought, The expression (16) for the addition of adaptive control weights shows:

(16) $$w=\left(w_{start}-w_{final}\right)*{(1-\left(\frac{t}{T}\right)}^{r})+w_{final}$$

Where $w_{start}$, $w_{final}$ are the maximum and minimum values of the control weights, they usually go to 0.9 and 0.1. r is the degree of bending of the control adaptive weight curve, which is usually taken as an integer as needed.

(5) Variation: To guarantee that the algorithm is capable of conducting a comprehensive global search during the later iterations, the zebra population is perturbed by the normal variation operator with the expression shown in (17):

(17) $$X_{new}^{j}=X_{origin}^{j}+N\cdot X_{origin}^{j}$$

where $X_{new}^{j}$ denotes the location of the mutated zebra, $X_{origin}^{j}$ is the location of the original zebra, and N is a randomly standardized normally distributed number obeying a mean of 0 and a variance of 1.

### 3.2 ELM neural network

ELM is a feed-forward neural network that comprises input, hidden, and output layers. The connection weights and thresholds of the input and hidden layers are assigned randomly, while the weights of the hidden and output layers are determined through least squares. Therefore, the ELM network demonstrates rapid training speed and exceptional generalization capabilities. In this research, our objective is to further enhance the predictive accuracy of the ELM network. To achieve this, we introduce the Improved Zebra Optimization Algorithm to optimize the connection weights and thresholds within the input and hidden layers. This optimization process results in the development of a more accurate ELM prediction model.

### 3.3 Phase space reconstruction

Phase space reconstruction stands as one of the fundamental theories in the realm of chaotic time series prediction. This technique plays a crucial role by mapping the original one-dimensional data into a higher-dimensional space. By doing so, it effectively captures and reveals more intricate and useful information from the data, as referenced in Ref. [29]. By using the C–C method [30], we calculate the embedding dimension $m_{1}$ and the delay time $\tau_{1}$ of the phase space reconstruction, which learns the nonlinear relationship between the sea clutter data by ELM, and the number of input nodes of the ELM network is derived from the literature [31], given as $m_{1}\tau_{1}$.

### 3.4 Steps for detecting small targets at low altitude over the sea surface

Step 1 Sea clutter pre-processing:

(I) The original sea clutter signal is CEEMDAN decomposed to obtain a series of IMFs;
(II) Obtain the RCMDEs for a series of IMFs and divide them into noise-dominated, signal-noise-mixed, and signal-dominated depending on the decision criterion;
(III) The noise-dominated IMF component is denoised with adaptive wavelet thresholding. The IMF component of the signal-noise mixed is denoised with SG filtering. And no change is made to the signal-dominated IMF component;
(Ⅳ) Reconstruct the denoised sea clutter signal by adding the denoised and unprocessed IMFs;

Step 2 Modeling of optimal sea clutter prediction:

(I) Normalizing the sea clutter signal in the range of [-1,1], the specific processing expression is as (18):

(18) $$x^{\prime }=2*\frac{x-x_{\min}}{x_{\max}-x_{\min}}-1$$

(II) Reconstructing the phase space from the embedded dimensions and delay times of the above-normalized sea clutter using the C–C method;
(III) Take $m_{1}\tau_{1}$ as an input to the ELM network and divide the training and test sets;
(Ⅳ) The training set is substituted into ELM and the initial weights and thresholds of the network are optimized using IZOA to obtain the best IZOA-ELM prediction model;

Step 3 Low-altitude small target detection:

(Ⅰ) Substitute the test set {y(i),i=1,2,...,N} into the optimal IZOA-ELM prediction model to obtain the prediction value $\hat{y}\left(i\right)$. The prediction error $error\left(i\right)=\left|y\left(i\right)-\hat{y}\left(i\right)\right|$.

is obtained, and low-altitude small target detection is performed based on the error;

(II) The RMSE is obtained and the denoising effect is evaluated according to the size of the RMSE before and after denoising, the expression is as (19):

(19) $$RMSE=\sqrt{\frac{1}{N}\sum_{i=1}^{N}\text{err}or^{2}\left(i\right)}$$

**Fig. 2:**
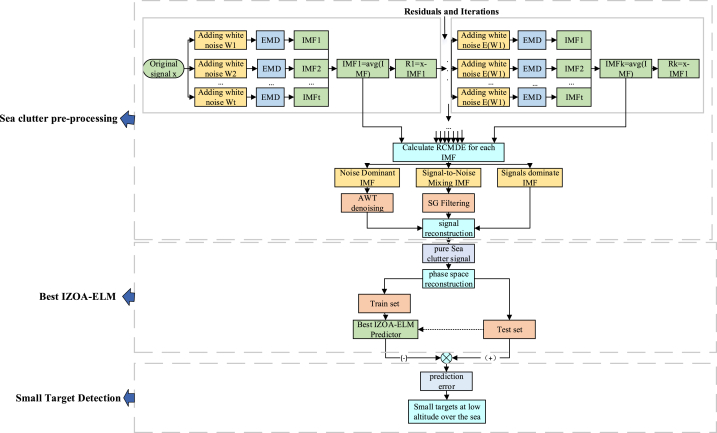
Flowchart of detection of low altitude small targets on sea surface.

The detailed flowchart result is shown in Fig. 2:

## 4 Simulation experiment

### 4.1 Source of data

To assess the practical effectiveness of our small target detector operating at low altitudes amidst sea clutter, we rely on the integration of CEEMDAN, IZOA, and ELM. We use the 1993 IPIX radar measured sea clutter data from McMaster University, Canada [32] as well as the latest phase of the SPPR radar measured sea clutter data released by Naval Aeronautical University in 2022 [33].In the context of the HH polarization mode, a total of 14 distinct sea conditions have been carefully chosen. Each of these conditions comprises 14 distance cells, and within each distance cell, a comprehensive dataset is collected, consisting of 131072 sampling points. The main parameters of the IPIX radar data are shown in Table 1. And the SPPR radar sea clutter data is also selected HH polarization mode, mainly study the T2 pulse echo signal, which has 1000 distance cells, each distance cell has 131072 sampling points, the 2 sets of data selected are shown in Table 2.

**Table 1:** Main parameters of IPIX radar data.

Data number	primary target unit	Sub-target units	Wind speed (km/h)	Wave height (m)
#17	9	8:11	9	2.2
#26	7	6:8	9	1.1
#30	7	6:8	19	0.9
#31	7	6:9	19	0.9
#40	7	5:8	9	1.0
#54	8	7:10	20	0.7
#280	8	7:10	10	1.6
#310	7	6:9	33	0.9
#311	7	6:9	33	0.9
#320	7	6:9	28	0.9

**Table 2:** Main parameters of SPPR radar data.

Data number	Target unit	Sea state	Wave height(m)
20221112171210_stare_HH	667–677	4	1.8
20221115060032_stare_HH	673–681	2	0.4

### 4.2 Measured sea clutter background prediction accuracy comparison

In order to provide a comprehensive assessment of the prediction accuracy achieved by the IZOA-ELM network, we have conducted comparative experiments. For this purpose, we have selected a set of well-established machine learning models, including RBF, SVM, LS-SVM, ELM, and LSTM. These models serve as benchmarks for a clear and rigorous evaluation of the performance of the IZOA-ELM network. In this experiment, we have specifically chosen the pure sea clutter unit labeled as #17, devoid of any target, to assess the performance of the network described in this paper. Under identical experimental conditions, we are utilizing 800 sampling points to calculate the Root Mean Square Error as a key metric for evaluating the network's effectiveness.

Observing the data presented in Table 3, the IZOA-ELM network introduced in this paper exhibits notably high prediction accuracy, particularly concerning pure sea clutter. This performance underscores the network's capability to precisely anticipate the intricate and dynamic variations within sea clutter, leveraging its inherent chaotic properties. Such accurate predictions pave the way for potential small target detection by utilizing prediction errors in subsequent phases.

**Table 3:** RMSE for each network.

network	RBF	SVM	LS-SVM	ELM	LSTM	IZOA-ELM
RMSE	0.0429	0.0292	0.0193	0.0019	0.0014	0.0010

### 4.3 Empirical testing of IZOA-ELM detector generalization in a sea clutter background

**Fig. 3:**
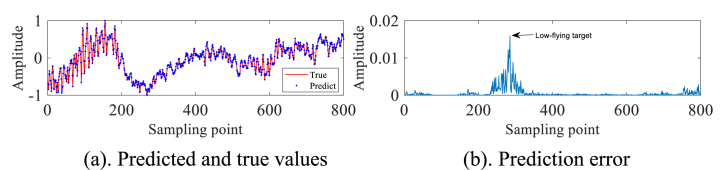
Primary target unit for #320.

In order to validate the practical utility of the IZOA-ELM detector in the real-world scenario of detecting small targets at low altitudes amidst actual sea clutter, We selected IPIX radar and SPPR radar measured sea clutter data for our experiments. As a preliminary step in our experiment, we have processed the pure sea clutter unit labeled as #320, excluding any target information. Subsequently, a subset of 2000 sampling points has been chosen for further analysis and experimentation. These 2000 data are first normalized and then phase space reconstruction is performed by the C–C method to obtain $m_{1}=3$ and $\tau_{1}=10$. A total of 1000 data sets have been selected for the purpose of creating a training set. These selected data sets are then utilized within the framework of an ELM. IZOA performs optimization to obtain the best prediction model IZOA-ELM. Subsequently, the main target unit (#320) comprising both the target and sea clutter is further processed. Specifically, 900 sampling points have been meticulously chosen from this subset to undergo the aforementioned process. Construct the phase space based on the above $m_{1}$, $\tau_{1}$ and pick 800 data sets as the test set. The experimental findings are depicted in Fig. 3a and b. In particular, a substantial error is evident at the 280th sampling point, whereas the errors at other points are relatively minimal. This leads us to conclude that small low-altitude targets are likely present in the area corresponding to the 280th sampling point.

**Fig. 4:**
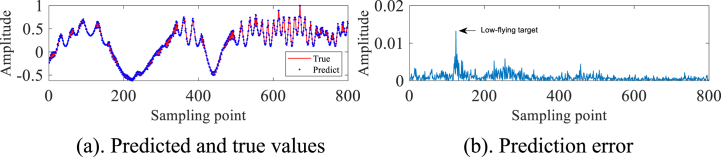
Primary target unit for #40.

**Fig. 5:**
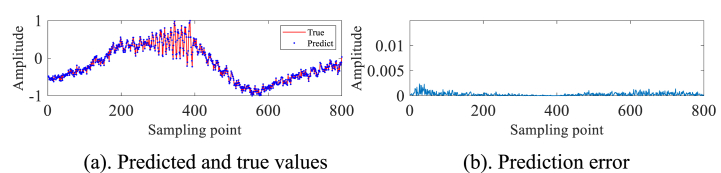
Primary target unit for #17.

**Fig. 6:**
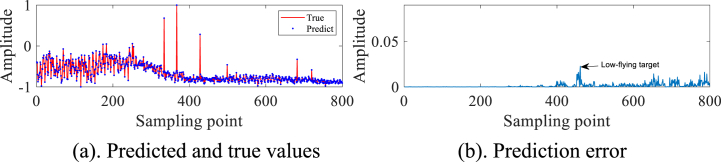
Primary target unit for 20221115060032_stare_HH.

In order to avoid the chance of the experimental results, we continue to select the #40 and #17 sets of data of IPIX and the 20221115060032_stare_HH data of SPPR for validation, and obtain the results as shown in Fig. 4a, 4b、 Fig. 5a and b and Fig. 6a and b. Upon analysis of the figure, it is apparent that within data set #40, a substantial error is evident at sampling point 110. This leads us to infer the potential existence of a low-altitude small target at this specific location. Remarkably, this observation aligns with the expected characteristics of sea clutter present in group #40. In contrast, when we examine data set #17, we observe that the prediction error amplitudes are predominantly small. Furthermore, the disparities between these errors are not substantial enough to confidently identify the presence of small targets at low altitudes. Besides, this method roughly detects the small target in 20221115060032_stare_HH, but it is more affected by the surrounding noise due to no denoising operation.

In summary, the experiments reveal that the IZOA-ELM detector demonstrates a notable ability to detect small targets at low altitudes across a majority of the sea clutter data. It is important to note, however, that the method's efficacy may be compromised in instances where the measured sea clutter data is significantly influenced by surrounding noise.

### 4.4 Low-altitude small target detection based on CEEMDAN-IZOA-ELM method

Given that the sea clutter data from set #17 exhibits a notable influence from surrounding noise, it is imperative to apply a denoising process before incorporating this data into subsequent analyses or experiments. The initial step involves selecting the pure sea clutter data (without any target) from dataset #17 for denoising. Subsequently, the trained IZOA-ELM detector for pure sea clutter is obtained by following the procedures detailed in section 4.3 And then the following is the denoising of the #17 main target unit, the steps are as follows.

**Fig. 7:**
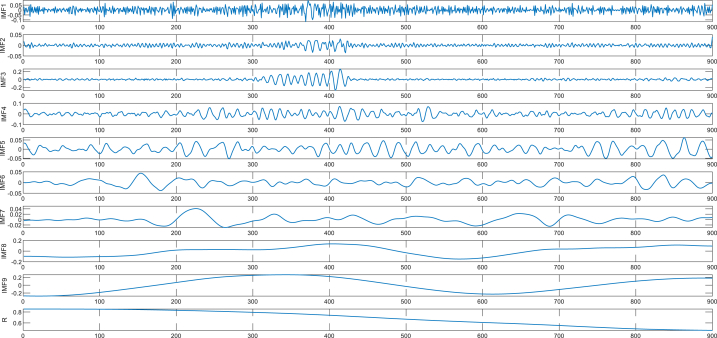
Main target unit of #17 decomposed by CEEMDAN.

For the main target unit #17, 900 sampling points were selected and decomposed using CEEMDAN to obtain a series of IMF and residual components. As shown in Fig. 7, the last IMF represents the residual component. Fig. 7 provides a clear illustration of the CEEMDAN decomposition process, which effectively dissects the original signal into a series of IMFs spanning a spectrum from high to low frequencies. These IMFs encapsulate both the noise and the valuable signals inherent in the original signal. Therefore, it is particularly important to find the boundary between noise and signal.

**Fig. 8:**
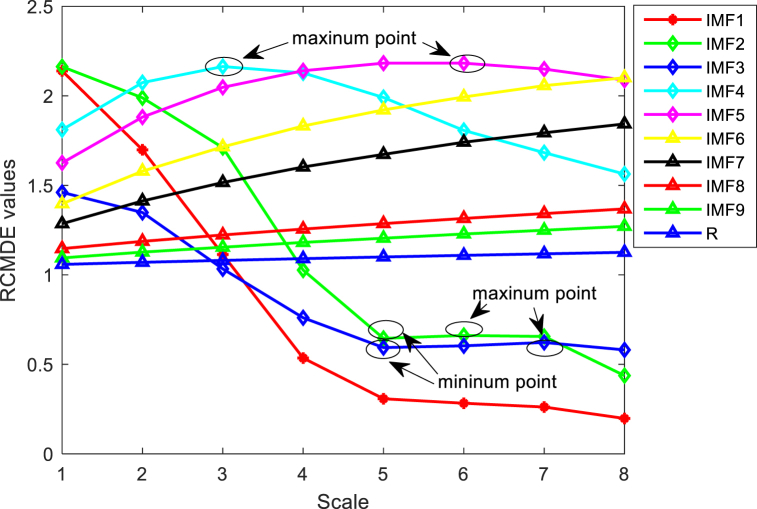
RCMDE folded spectrum for #17 main target unit.

Calculate RCMDE values for each of the aforementioned IMFs and generate folded spectra. As observed in Fig. 8, the RCMDE of IMF1 exhibits a consistently decreasing trend with notable upper and lower amplitude fluctuations, indicative of susceptibility to internal noise influence, categorizing it as a noise-dominated IMF. In contrast, IMFs 2 to 5 display RCMDE profiles containing extreme points, leading to their classification as signal-noise mixed IMFs. Finally, IMFs 6 to 9 and the residuals (R) all demonstrate a steady increasing trend with minor amplitude fluctuations, establishing them as signal-dominated IMFs.

**Fig. 9:**
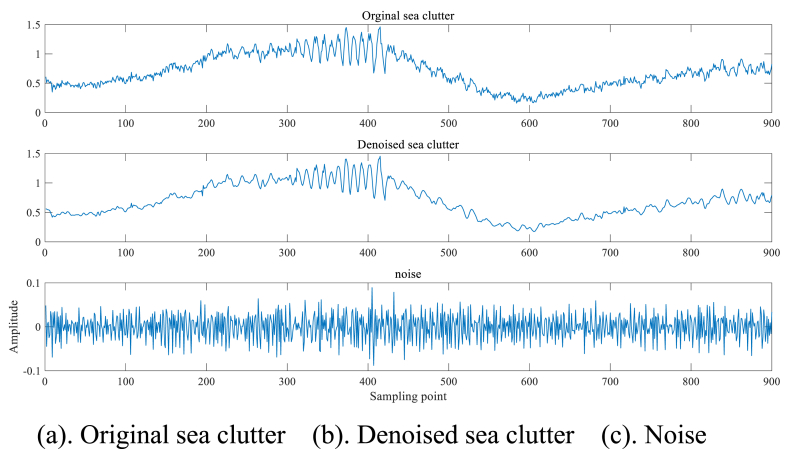
Signal after denoising of #17 sea clutter main target unit.

In the denoising process, AWT is applied to IMF1, while IMF2 to IMF5 are subjected to SG filtering. IMF6 to IMF9 and the residual R remain unaltered. The combination of these three categories of IMFs results in the reconstructed denoised sea clutter signal, as illustrated in Fig. 9a, b, 9c.

**Fig. 10:**
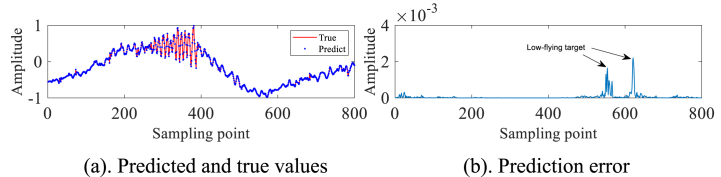
#17 Main target unit after denoising.

The denoised sea clutter signal is subsequently reconstructed in phase space using the previously derived values for $m_{1}$ and $\tau_{1}$, resulting in the creation of 800 data sets for prediction. These data sets are then input into the pre-trained IZOA-ELM detector. The resulting outcomes are visually represented in Fig. 10a and b, revealing the presence of two small, low-altitude targets.

**Fig. 11:**
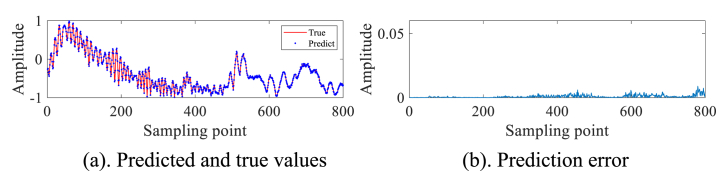
#280 Main target unit before denoising.

**Fig. 12:**
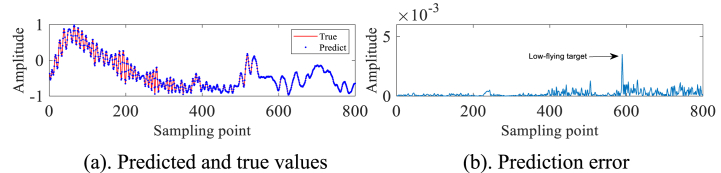
#280 Main target unit after denoising.

**Fig. 13:**
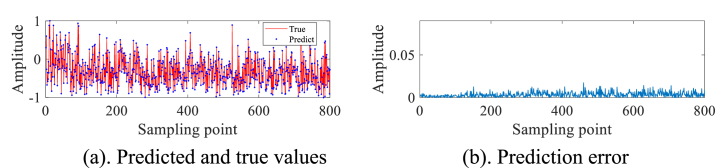
20221112171210_stare_HH target unit before denoising.

**Fig. 14:**
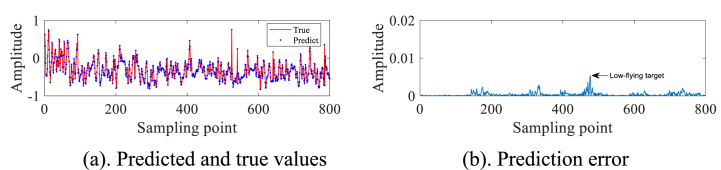
20221112171210_stare_HH target unit after denoising.

To further verify the effectiveness of the algorithm, the #280 measured sea clutter data of IPIX and the 20221112171210_stare_HH measured sea clutter data of SPPR are selected for the above operation. The experimental results are presented in Fig. 11a, 11b、 Fig. 12a, 12b、 Fig. 13a, 13b、 Fig. 14a and b. As can be seen from the above figures, before denoising #280 and 20221112171210_stare_HH target cells are not detected as small targets. And after denoising by this method, #280 detects a small target at point 600, while 20221112171210_stare_HH detects one at point 500.

**Fig. 15:**
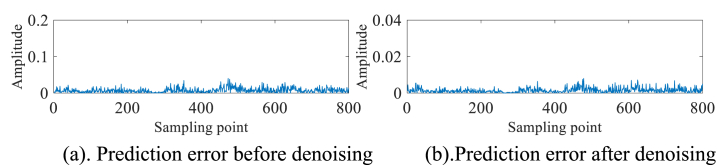
#17 Pure sea clutter unit.

**Fig. 16:**
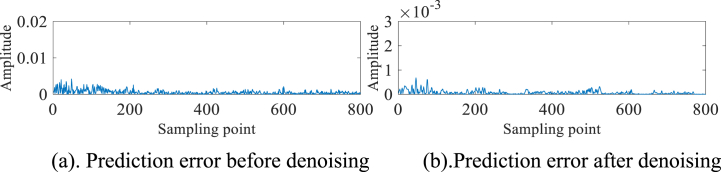
#280 Pure sea clutter unit.

In order to mitigate the generation of false targets subsequent to denoising the pure sea clutter data (devoid of any targets), it is imperative to compare the prediction errors both before and after the denoising process. As part of our validation procedure, we selected an additional set of pure sea clutter units, which were free from any target presence, from the original 14 distance units. We aimed to establish the consistency of the pure sea clutter units before and after denoising by conducting a comparative analysis of the prediction errors both prior to and following the denoising process. The fluctuation of the prediction error magnitude before and after denoising should be small because the chaotic properties of the pure sea clutter unit are not destroyed by the small target. Upon careful examination of Fig. 15a and b and Fig. 16a and b, it becomes evident that the amplitude fluctuations of prediction errors prior to and subsequent to denoising the pure ocean clutter cells, devoid of any targets, do not exhibit significant magnitudes. By virtue of the inherent characteristics of the pure sea clutter unit, no instances are observed in which the amplitude exhibits significant magnitudes at specific sampling points.

Through the rigorous examination of multiple sets of measured sea clutter data, it has been ascertained that the CEEMDAN-AWT-SG method proposed in this paper is proficient in successfully eliminating noise interference while preserving the inherent chaotic properties of sea clutter. The denoising effect has been quantified through a comparative analysis of RMSE values before and after the denoising process, as presented in Table 4. The results demonstrate a noteworthy enhancement, with the RMSE values after denoising exhibiting an improvement of at least one order of magnitude in comparison to their pre-denoising counterparts.

**Table 4:** RMSE before and after denoising.

batch number	pre-denoising	denoising
#17	0.0012	2.07e-04
#280	0.0014	3.46e-04
20221112171210_stare_HH	0.0045	6.49e-04

## 5 Conclusion

When detecting low-altitude small targets based on the intrinsic characteristics of sea clutter, the received sea clutter signals are frequently subject to interference from surrounding noise. This interference undoubtedly augments the complexity of subsequent small target detection efforts. So this paper proposes a CEEMDAN-AWT-SG denoising algorithm combined with an IZOA-ELM detector. The initial noisy sea clutter data undergoes decomposition into a series of IMFs using CEEMDAN. Subsequently, the RCMDEs of these IMFs are calculated and visualized in the form of folded spectra. The RCMDE folds of the IMF component with a higher level of noise exhibit a consistent monotonic decrease while displaying fluctuations in magnitude. Conversely, the RCMDE folds of the IMF component resulting from the signal-to-noise mixing lack monotonicity and display the presence of one or more extreme points. The RCMDE folds of the IMF component dominated by the signal displays a pronounced, monotonically increasing trend, indicative of its substantial signal share. When applying AWT for denoising the noise-dominated IMF component, it preserves a minor portion of valuable signal content while effectively eliminating a significant amount of noise. In the context of signal-to-noise mixing, the utilization of SG filtering demonstrates an effective capability to eliminate noise interference and enhance the smoothness of the IMF component. The denoised and unprocessed IMF components are combined to reconstruct the denoised sea clutter signal. Experiments conducted on two distinct groups of measured sea clutter, labeled as #320, #40 and 20221115060032_stare_HH, have revealed that in scenarios where sea clutter is less affected by noise, the IZOA-ELM detector is capable of direct detection of small targets at low altitudes. The methodology was subsequently tested on datasets labeled #17, #280, and 20221112171210_stare_HH, characterized by higher levels of noise interference, and the detection process proved unsuccessful in these cases. At this stage, the sea clutter data undergoes denoising through the CEEMDAN-AWT-SG method, as proposed in this paper, followed by the detection process using the IZOA-ELM detector. The experimental results demonstrate the method's effectiveness in noise removal from the original sea clutter while preserving its inherent chaotic characteristics. The RMSE exhibited a notable improvement of one order of magnitude before and after denoising, and the effective detection of small, low-altitude targets was achieved through the analysis of prediction errors.

## Data availability statement

All data used in this article was obtained from the following websites: http://soma.ece.mcmaster.ca/ipix/dartmouth/index.html.

https://radars.ac.cn/web/data/getData?dataType=DatasetofRadarDetectingSea_en&pageType=en.

## CRediT authorship contribution statement

**Shang Shang:** Writing – review & editing, Writing – original draft, Validation, Supervision, Funding acquisition. **Jian Zhu:** Writing – review & editing, Writing – original draft, Validation, Software, Resources. **Qiang Liu:** Writing – review & editing, Validation, Software. **Yishan Shi:** Writing – review & editing, Validation, Software. **Tiezhu Qiao:** Writing – review & editing, Validation, Software.

## Declaration of competing interest

The authors declare the following financial interests/personal relationships which may be considered as potential competing interests:Shang Shang reports administrative support, article publishing charges, statistical analysis, and writing assistance were provided by 10.13039/501100001809National Natural Science Foundation of China. Jian Zhu reports article publishing charges and statistical analysis were provided by Jiangsu Provincial Graduate Student Research and Practice Innovation Program Funded Projects. If there are other authors, they declare that they have no known competing financial interests or personal relationships that could have appeared to influence the work reported in this paper.
